# Effects of intranasal oxytocin on neural reward processing in children and adolescents with reactive attachment disorder: A randomized controlled trial

**DOI:** 10.3389/frcha.2022.1056115

**Published:** 2023-01-10

**Authors:** Shinichiro Takiguchi, Kai Makita, Takashi X. Fujisawa, Shota Nishitani, Akemi Tomoda

**Affiliations:** ^1^ Department of Child and Adolescent Psychological Medicine, University of Fukui Hospital, Fukui, Japan; ^2^ Division of Developmental Higher Brain Functions, United Graduate School of Child Development, University of Fukui, Fukui, Japan; ^3^ Research Center for Child Mental Development, University of Fukui, Fukui, Japan

**Keywords:** reactive attachment disorder (RAD), intranasal oxytocin, functional magnetic resonance imaging (fMRI), reward, childhood maltreatment, randomized controlled trial

## Abstract

Reactive attachment disorder (RAD) is associated with socially and emotionally withdrawn/inhibited behaviors and reduced neural responses to rewards. Children and adolescents with RAD show aberrant attachment behaviors, and existing psychotherapies are difficult to maintain; therefore, pharmacological interventions to aid and boost treatment responses are needed. Oxytocin (OT) administration is known to promote reward functioning. We investigated whether single-use intranasal OT administration improved neural responses during reward processing in patients with RAD compared with healthy controls. Twenty-four male children and adolescents with RAD (10–18 years old) and 27 age- and sex-matched typically developing individuals (10–17 years old) were included in this randomized, double-blind, placebo-controlled, cross-over, functional magnetic resonance imaging study. Following a single intranasal OT (24 IU) or placebo administration, neural responses were investigated using a monetary reward task. In the RAD group, OT significantly increased subjective motivation scores, significantly enhanced activation in the right middle frontal gyrus, and reduced activation in the right precentral gyrus during the monetary reward task. Additional analyses revealed increased activation in the bilateral caudate at a more lenient threshold. Under placebo conditions, the severity of internalizing problems in patients with RAD was negatively correlated with ventral striatal activity. Moreover, the effect of OT on ventral striatum activity was positively associated with the severity of internalizing problems in patients with RAD. Intranasal OT administration enhanced activity in the reward pathway in male children and adolescents with RAD, suggesting that exogenous OT promotes reward processing and reward-related motivational behavior in these individuals. Further investigation is needed to fully understand the neural mechanisms of intranasal OT and identify novel targets for pediatric cases with RAD.

**Clinical trial registration:** UMIN-CTR; UMIN000013215. URL: https://upload.umin.ac.jp/cgi-open-bin/ctr/ctr_view.cgi?recptno=R000015419

## Introduction

Childhood maltreatment (CM) and neglect are critical risk factors for reactive attachment disorder (RAD), which is characterized by socially and emotionally withdrawn/inhibited and aberrant attachment behaviors, with a failure to seek and respond to comfort (1), affecting approximately 1% of the general population (2, 3). In children and adolescents, RAD is associated with internalizing problems (4, 5), and multiple psychiatric comorbidities [i.e., depression, anxiety, and substance use disorders (SUDs)], which are common and persist into adulthood (6–10).

Appropriate interventions for children and adolescents with RAD may reduce long-term adverse outcomes and promote adaptive recovery. The most important intervention is the development of a secure attachment between children and their caregivers by enabling positive interactions between them (11, 12), which is associated with decreased engagement in high-risk behaviors, few mental health problems, and enhanced social skills and coping strategies (13), possibly preventing the intergenerational transmission of CM. However, children with RAD often lack well-formed attachments with their caregivers, and highly stressed caregivers find it difficult to adequately and sensitively respond to the child's angry or anxious feelings and develop a strong attachment with them (14). Although pharmacological interventions are indicated to treat comorbidities associated with RAD, no medication has been recommended to treat the core features of RAD (14). Adverse endocrine and metabolic effects of psychotropic medications seem prevalent in children and adolescents (15). Thus, pharmacological intervention trials are needed with safety monitoring and low risks of adverse effects.

While the underlying pathogenesis of RAD remains to be completely elucidated, our recent neuroimaging studies in children and adolescents with RAD indicate alterations in reward circuitry functioning (16, 17) and pathways mediating emotion regulation (18–20). In a former study, a voxel-based morphometry analysis of magnetic resonance imaging (MRI) images revealed increased gray matter volume in the pallidum and thalamus (20). Moreover, our previous functional MRI (fMRI) studies revealed reduced ventral striatum activity during a monetary reward task and its negative relationships with post-traumatic stress symptom severity or avoidance attachment scores (16). Therefore, therapeutic interventions addressing the neural dysfunctions in reward processing that may be implicated in the clinical symptoms of RAD may improve reward functioning and treatment outcomes.

Recent evidence supporting the intranasal administration of oxytocin (OT) for promoting trust, prosocial behavior, and approach behavior in healthy individuals (21) has made exogenous OT a potential candidate for an effective intervention to treat psychiatric disorders, especially in conditions characterized by aberrant reward-related motivational behaviors (22). Recent studies on children with autism spectrum disorder (ASD) indicate that intranasal OT administration is generally well tolerated with few side effects (23–26). Increasing evidence supports the important role of neuropeptide OT in regulating mother-infant bonding and attachment in humans (27, 28), and atypical OT levels in plasma, cerebrospinal fluid, and saliva have been found in children subjected to maltreatment and lacking attachment or bonding with a primary caregiver (29–34). OT acts both as a neurotransmitter and neuromodulator, regulating neuroendocrine, psychophysiological, and socioemotional responses in animals and humans (22, 28, 35, 36). OT neurons centrally project to brain regions involved in reward processing and reinforcement, as well as to those involved in the manifestation of social behaviors. These regions include the ventral striatum—including the caudate and nucleus accumbens (NAcc)—and the prefrontal cortex, where OT receptors are also distributed (37). Further, OT facilitates motivated behavior and social interaction by modulating dopaminergic activity within mesocorticolimbic salience dopamine reward networks (38, 39).

In fMRI studies on healthy participants, intranasal OT administration increased the neural responses in reward pathway regions, such as the striatum and ventral tegmental area, during reward and punishment anticipation, and in the presence of positive stimuli (40–43). SUDs are also reportedly more prevalent in children with disrupted attachment who experienced early maternal withdrawal (9). Relative to placebo (PLC), intranasal OT administration enhanced behavioral responses to monetary rewards in cocaine-dependent patients (44). Exogenous OT has also enhanced neural responses to monetary incentives in the putamen, insula, and anterior cingulate cortex in patients with post-traumatic stress disorder (PTSD) (45), which has been classified as a trauma- and stressor-related disorder, similar to RAD.

Childhood and adolescence are characterized by marked changes in psychological development, brain sensitivity to rewards and incentives, neuroendocrine function, and shifting relationships between children and their caregivers and peers (46). Child-caregiver interaction modulates fundamental brain processes (47, 48), whereas socializing with peers drives more complex psychological development (49). Intranasal OT may modulate the developing brain, advancing the reorganization of functional reward networks during childhood and adolescence in individuals with RAD.

Investigating the neurobiological effects of intranasal OT administration on reward processing in RAD may provide valuable insights into the application of OT. Thus, we investigated the effects of intranasal administration of a single dose of OT on neural reward processing in children and adolescents with RAD (RAD group) and typically developing (TD) children and adolescents (TD group/control group) during a monetary reward task (50). We hypothesized that OT would increase activation in the reward pathway during motivational processing in the RAD group. Based on our previous findings of ventral striatal dysfunction in individuals with RAD (16, 17), we expected a difference in activity patterns in the caudate nucleus and NAcc in the RAD group, relative to the TD group, during the monetary reward condition. The effects of intranasal OT may depend on inter-individual and social contextual factors (35, 51). Furthermore, we explored the relationships between neural responses to OT administration and behavioral problems in children and adolescents with RAD. For this purpose, plasma OT was measured simultaneously to confirm that nasal OT reliably circulated in the blood. Thus, it allowed us to test the further hypothesis that rewards system activity is associated with plasma OT concentration that is enhanced by the external administration.

## Materials and methods

### Participants

Twenty-nine male children and adolescents with RAD (aged 10–18 years) and 28 age- and sex-matched TD (aged 10–17 years) individuals were included in the randomization procedure. Several participants were excluded from the analysis because they dropped out at the second visit owing to mental and physical fatigue during the MRI scans (RAD group, *n* = 3) or low scan data quality (RAD group, *n* = 2; TD group, *n* = 1). Ultimately, 24 participants from the RAD group (mean age, 13.3 years) and 27 participants from the TD group (mean age, 13.0 years) were included in the final analysis.

We recruited children and adolescents with RAD from the Department of Child and Adolescent Psychological Medicine at the University of Fukui Hospital. The diagnosis of RAD was assessed by two board-certified child psychiatrists (first and fifth authors of this manuscript) based on the criteria of the Diagnostic and Statistical Manual of Mental Disorders, Fifth Edition (DSM-5) (1). To exclude other psychiatric diseases (e.g., PTSD, mood disorders, anxiety disorders, and SUDs) and neurodevelopmental disorders such as ASD or attention-deficit hyperactivity disorder, the Mini-International Neuropsychiatric Interview for Children and Adolescents (52) was administered. All participants with RAD had a history of CM, including physical abuse, neglect, emotional abuse, and sexual abuse (Table 1). Fourteen participants with RAD were medication-naïve (58.3%), while 10 participants received medication, including osmotic-release oral system methylphenidate (*n* = 2); atomoxetine (*n* = 5); aripiprazole (*n* = 2); risperidone (*n* = 1); clotiazepam (*n* = 1); valproate acid (*n* = 1); or carbamazepine (*n* = 1) for aggressive behavior and irritability or age-inappropriate inattention and impulsivity, followed by a wash-out period ≥72 h (five times the half-life) before all procedures including the fMRI examinations.

**Table 1:** Demographic and clinical characteristics of the participants.

	TD group	RAD group	*p*
	*n* = 27	*n* = 24	
Age (years), Mean (SD)	13.0 (1.6)	13.3 (1.9)	0.58
Male participants, n (%)	27 (100)	24 (100)	1.00
Handedness (R/L)	25/2	21/3	0.34
Types of maltreatment, *n* (%)			
Physical abuse	–	16 (67)	
Emotional abuse	–	22 (92)	
Neglect	–	17 (71)	
Sexual abuse	–	4 (17)	
Number of types of maltreatment, Mean (SD)	–	2.6 (0.9)	
Duration (years) of maltreatment, Mean (SD)	–	8.6 (4.4)	
WISC-IV FSIQ, Mean (SD)	107.7 (9.3)	93.7 (9.5)	<0.001
SDQ Total difficulties score, Mean (SD)	5.4 (4.2)	14.3 (7.3)	<0.001
Internalizing problems, Mean (SD)	1.9 (1.9)	6.0 (3.7)	<0.001
Externalizing problems, Mean (SD)	3.4 (2.8)	8.3 (4.5)	<0.001
DSRSC, Mean (SD)	7.5 (5.3)	12.8 (6.5)	0.003
CATS, Mean (SD)	5.8 (6.2)	41.3 (22.7)	<0.001

TD, typically developing; RAD, reactive attachment disorder; SD, standard deviation; R/L, right/left; WISC-IV, Wechsler Intelligence Scale for Children-Fourth Edition; FSIQ, Full-Scale Intelligence Quotient; SDQ, Strengths and Difficulties Questionnaire; DSRSC, Depression Self-Rating Scale for Children; CATS, Child Abuse and Trauma Scale.

TD children and adolescents were recruited from local communities through advertisements. The inclusion criteria included a lack of history of CM as assessed using the Adverse Childhood Experience Questionnaire and not meeting any DSM-5 criteria for psychiatric disorders (1). The exclusion criteria for all participants included contraindications for MRI; a Full-Scale Intelligence Quotient (FSIQ) <80 on the Wechsler Intelligence Scale for Children-Fourth Edition (WISC-IV) (53) or Wechsler Adult Intelligence Scale-Third Edition (WAIS-III) (54); any history of severe head trauma, drug or substance abuse, major physical or neurological illnesses, or medical conditions that could adversely affect growth and development; and excessive head motion (>6 mm/degrees) during the scanning.

### Study design and procedure

**Figure 1:**
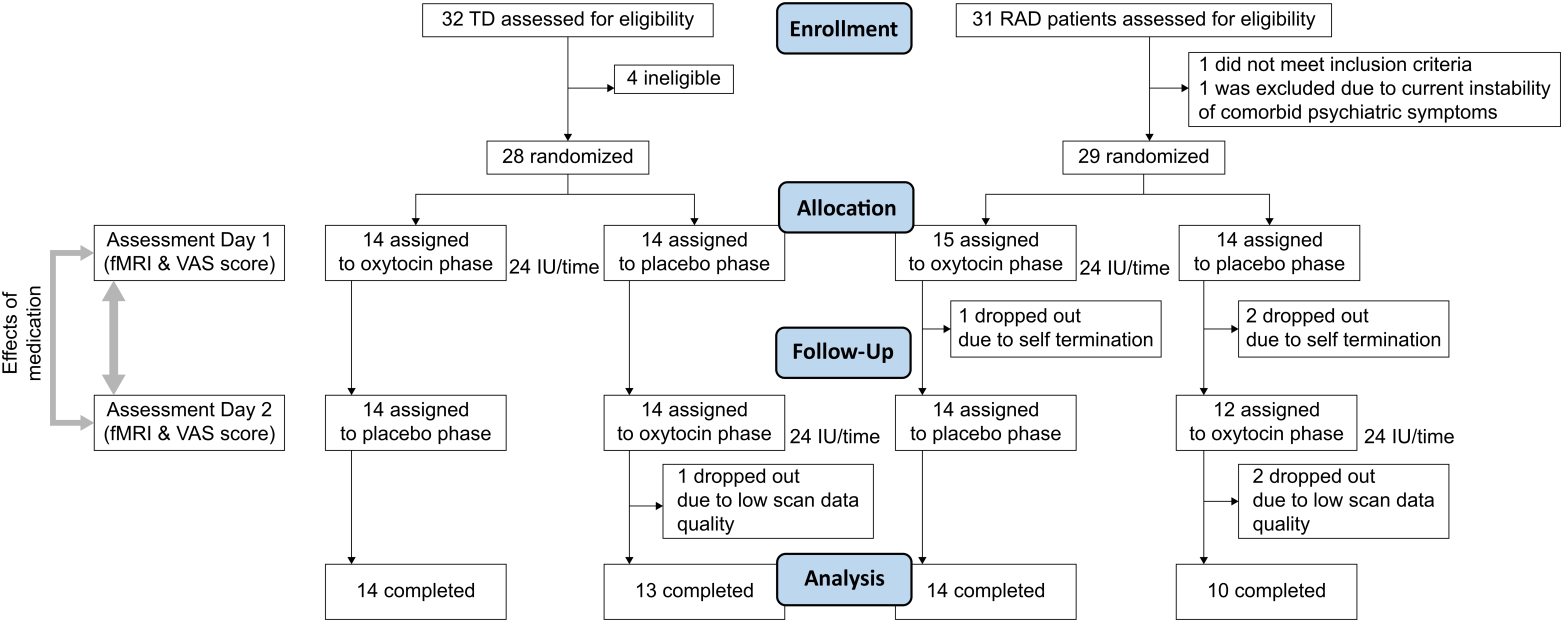
CONSORT flow diagram. VAS, visual analogue scale; TD, typically developing; RAD, reactive attachment disorder.

The required sample size of more than 20 participants per group (TD and RAD groups) was calculated using a statistical power analysis (55). This study was a randomized, double-blind, PLC-controlled, within-subject, cross-over trial conducted at the University of Fukui Hospital from August 2013 to March 2019 (Figure 1). The randomization schedule was generated by an unmasked statistician who was not involved in the trial or data analysis. Randomization was centralized using a computer-generated list. Participants and staff were masked to the treatments (OT vs. PLC), and the allocation sequence was not disclosed until all participants completed the trial. All participants received a single dose of intranasal OT (24 IU; Syntocinon spray, Novartis, Basel, Switzerland) or PLC (0.8% saline) in accordance with the recommendations for administration procedures (56) approximately 45 min before fMRI. Participants in both the TD and RAD groups participated in two MRI scanning sessions. The two scan sessions were scheduled at least 1 week apart to minimize the possible carryover effects of OT administration. Moreover, the second visit was scheduled according to the participants’ availability to avoid the stress involved with their school events and the physical discomfort of undergoing MRI (mean number of days between scans = 36.3 ± 37.1; TD group, 40.8 ± 28.2; RAD group, 31.2 ± 44.5; *p* = 0.366). Immediately after the MRI, participants recorded their subjective motivation on a visual analog scale (VAS) with values ranging from 0 (not at all) to 100 (entirely) during the monetary reward task.

The protocol for the present study was approved by the Research Ethics Committees of the University of Fukui (Approval No. 20138012) and conducted in accordance with the Declaration of Helsinki. All participants and their parent(s) or child welfare facility director provided written informed consent or assent for participation. This study was registered with the University Hospital Medical Information Network (UMIN000013215).

### Clinical symptom measures

Data on history of maltreatment were obtained from Child Protective Services. To assess the perceived severity and types of maltreatment, the Child Abuse and Trauma Scale (CATS) (57) was administered to all participants. The clinical symptom measures were administered to the participants by board-certified child psychiatrists (first and fifth authors of this manuscript). The Depression Self-Rating Scale for Children (DSRSC) (58) was used to evaluate depressive symptoms. Parents or caregivers were asked to complete the parental version of the Strengths and Difficulties Questionnaire (SDQ) (59) to assess the severity of RAD-related emotional and behavioral symptoms. The SDQ is a 25-item parent-report questionnaire that assesses a child's internalizing (emotional and peer) and externalizing (conduct and hyperactivity) problems and behavioral difficulties, as well as prosocial behavioral tendencies (60). A positive association between scores on the SDQ and the Relationship Problems Questionnaire, which was developed to screen symptoms of RAD, has been previously reported (61).

### Measurement of plasma OT concentration

Whole blood samples were collected immediately before intranasal OT/PLC administration and the measurements were considered the baseline for all participants (TD, *n* = 27; RAD, *n* = 24). Multiple blood samples were only collected in the subpopulation (TD, *n* = 15; RAD, *n* = 11) that consented to blood sampling 30 min after intranasal administration for both experimental conditions (OT and PLC). Blood samples were collected into chilled 2 ml EDTA-2Na-treated vacutainer tubes containing aprotinin (NIPRO Co., Osaka, Japan) from the medial cubital vein and placed on wet ice. Samples were promptly centrifuged (1,600 × g at 4°C for 15 min), and the plasma fraction was obtained. All plasma samples were stored at −80°C until OT measurement. Plasma OT concentrations were determined using an enzyme immunoassay Oxytocin ELISA kit (Enzo Life Sciences, Inc., Farmingdale, NY, United States).

Briefly, plasma samples (500 μl per sample) were extracted using an Oasis PRiME HLB 96-well plate with 30 mg sorbent per well (SKU: 186008054, Waters Inc.) and evaporated at room temperature using compressed nitrogen. Each evaporated sample was reconstituted in 250 μl of assay buffer before OT measurement to provide sufficient sample volume to run each sample in duplicate wells (100 μl per well). Samples were assayed with a microplate reader (SPECTRA MAX 250, Molecular Devices, LLC) for the 96-well format according to the manufacturer's instructions. The mean intra- and inter-assay coefficients of variation were less than 10.0%.

### Monetary reward tasks

**Figure 2:**
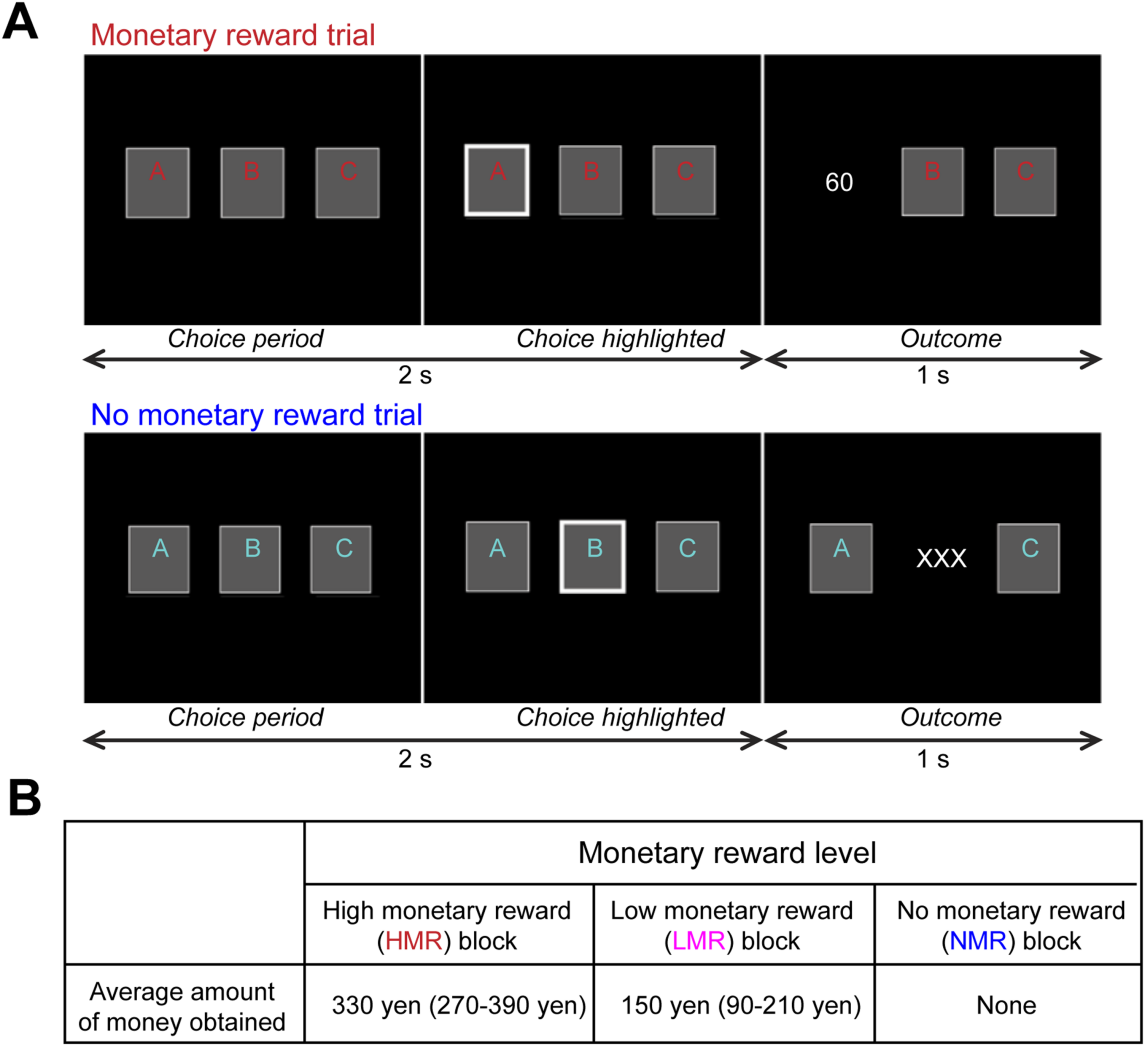
Experimental paradigm for the monetary reward task. The task and stimuli have been described in previous studies (16, 17, 50) as follows: (**A**) Each block consisted of eight trials with monetary reward or “no reward” conditions (24 s). Top: In each reward trial, participants were asked to choose one card within 2 s, and the outcome of the chosen card (0, 30, or 60 yen) was shown for 1 s. Bottom: In each “no reward” trial, participants were similarly asked to choose one card, although the outcome was always “XXX,” indicating no reward. For half of the participants, the colors (red and blue) used for the letters on the cards in the reward and “no reward” conditions were switched to control for differences in activity related to visual color processing. (**B**) Design of the monetary reward experiment. To manipulate monetary rewards, the amount of money each participant could earn in each block was predetermined.

Participants performed the block-design gambling task involving monetary rewards, which has elicited robust striatal responses in healthy individuals (16, 17, 50) (Figure 2, see Supplementary Materials and Methods). We asked participants to choose one of three cards by pressing a button. Each card was randomly associated with 0, 30, or 60 yen. Three conditions (high monetary reward [HMR], low monetary reward [LMR], and no monetary reward [NMR]) of eight trials (24 s) were performed. Unknown to the participants, the total reward was predetermined. In the HMR condition, participants earned an average of 330 yen. In the LMR condition, participants earned an average of 150 yen. In the NMR condition, the outcome was always “XXX” to control for effects other than reward level. The NMR or a fixation rest condition (24 s) was inserted between the two reward conditions. During MRI, participants performed four sessions consisting of four blocks from each of the four conditions (HMR, LMR, NMR, and fixation rest) lasting a total of 6 min 24 s (4 blocks × 4 conditions × 24 s per block).

### fMRI acquisition and analysis

The procedures for data acquisition, pre-processing, and first-level analysis were described in our previous study (17) (see Supplementary Materials and Methods). One individual from the TD group and two from the RAD group were excluded from the analyses due to low scan quality [excessive head motion >6 mm/degrees, as previously described in OT fMRI studies of adult patients (45, 62)]. In total, 27 TD individuals and 24 individuals with RAD were included for fMRI analyses.

All scans were acquired using a 3-T MR scanner (Discovery MR 750; General Electric Medical Systems, Milwaukee, WI, USA) with a 32-channel head coil (repetition time, 3,000 ms; echo time, 25 ms; flip angle, 90°; field of view, 192 mm; in-plane matrix size, 64 × 64 pixels, voxel dimensions, 3.0 × 3.0 × 3.0 mm; slice gap, 0 mm). Data were analyzed using SPM12 (The Wellcome Trust Centre for Neuroimaging, London, UK) implemented in MATLAB 2020a (Mathworks, Natick, MA, United States). Following realignment, all images were normalized to the SPM12 (echo planar imaging) image template. At the first level, individual task-related activation was evaluated. Three regressors for each condition (HMR, LMR, and NMR) were modelled at the onset of each block (duration, 24 s), which were convolved with a canonical hemodynamic response function to obtain the expected task-related signal change. The weighted sum of the parameters estimated during individual analyses consisted of “contrast” images. For the statistical analyses, motion parameters were modelled as regressors of no interest using the six parameters (three displacements and three rotations) obtained by the rigid-body realignment procedure.

Our previous studies using this gambling task revealed significantly reduced striatal activation in the RAD group compared with the TD group during the HMR condition (contrast, HMR > NMR) (16,17). Thus, we included this contrast in the present study. In the second-level, we analyzed contrast images corresponding to the HMR > NMR condition for group analyses using a random-effects model to obtain population inferences to identify brain regions that respond to monetary reward. We then compared activations between the TD and RAD groups in the OT and PLC conditions. A flexible factorial ANOVA was conducted to investigate the interactions between the between-subject factor group (TD/RAD) and the within-subject factor treatment condition (OT/PLC). In line with previous findings (16,17), we subsequently conducted a one-way, within-subject analysis of variance (ANOVA) to compare the activities and identify the main effect of OT administration within the TD and RAD groups separately under both conditions. Significant changes in signal for each contrast were assessed using *t*-statistics on a voxel-by-voxel basis. For the whole-brain analysis, the statistically significant threshold was set at *p* < 0.001 at the voxel level and family-wise error (FWE)-corrected *p* < 0.05 at the cluster level. The anatomical localization of significant clusters was investigated with the Automated Anatomical Labeling and Brodmann area (BA) atlases implemented in the MRIcron software package (63).

### Assessment of adverse effects of intranasal OT administration

In order to detect possible cardiovascular and pulmonary adverse effects caused by OT administration, we measured each participant's heart rate (HR), blood pressure (BP), and oxygen saturation *via* a pulse oximeter (SpO_2_) using a physiological monitoring system immediately before and 30 min after nasal spray administration. In addition, open-ended questions were used to assess medication side effects, such as headache, thirst, urination, wheezing, and chest pain.

### Statistical analyses

Demographic and clinical characteristics were compared between groups using the *χ*^2^ (chi-square), individual *t,* and Mann–Whitney *U* tests. The VAS scores and average reaction times (RTs) of all conditions were tested for the effects of group (TD/RAD) and treatments (OT/PLC) using repeated-measures ANOVA. For post-hoc tests, a Bonferroni's adjustment procedure was used. Statistical analyses were performed using SPSS software version 24 (IBM Corp., Armonk, NY, USA). Data were considered statistically significant at *p* < 0.05.

## Results

### Demographics and clinical characteristics

The clinical and demographic characteristics of the TD and RAD groups are shown in Table 1. Both groups were well-matched for age and handedness. Compared with the TD group, the RAD group showed a significantly lower FSIQ [*t* (49) = 5.17, *p* < 0.001] and significantly higher levels of perceived severity of maltreatment (CATS: *U* = 8.50, *p* < 0.001). Similarly, the RAD group had significantly higher levels of psychiatric symptoms: internalizing and externalizing behavioral problems (SDQ: *t* [33] = 4.73, *p* < 0.001 and *t* [37] = 4.52, *p* < 0.001, respectively) and depressive symptoms [DSRSC: *t* (48) = 3.13, *p* = 0.003].

### Plasma OT concentration

**Figure 3:**
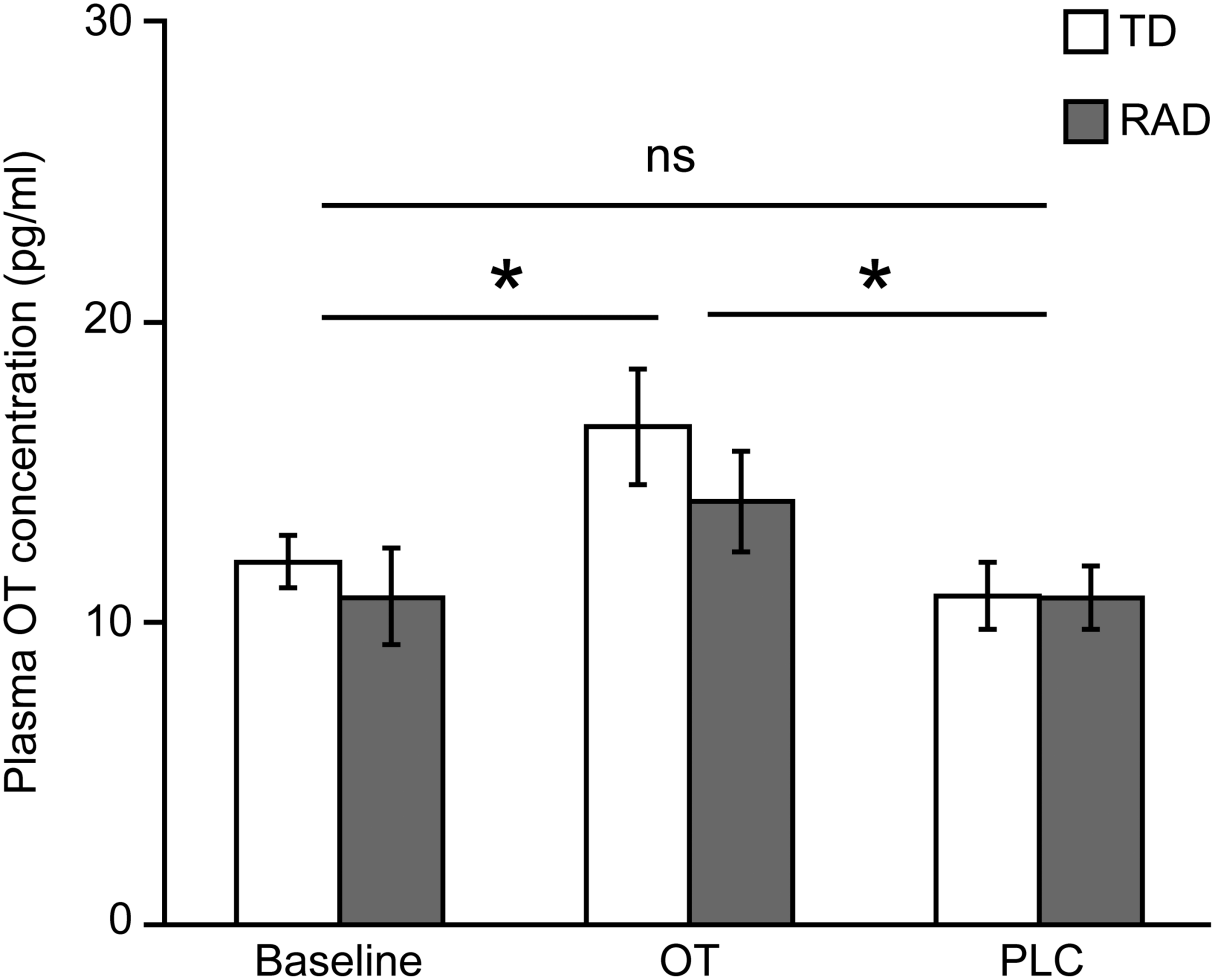
Plasma OT concentration. Plasma OT concentration at baseline, and after OT and PLC administration. The plotted values represent means and standard errors of the mean. *p < 0.05. OT, oxytocin; PLC, placebo; TD, typically developing; RAD, reactive attachment disorder. TD group (*n* = 15), RAD group (*n* = 11).

A two-way ANOVA was conducted to examine the effect of group (TD/RAD) and treatments (OT/PLC) on plasma OT concentration. There was a significant main effect of OT administration [*F* (2, 48) = 5.64, *p* = 0.006], whereas the main effect of group and their interactions were not significant (*F* [1, 24] = 0.83, *p* = 0.370 and *F* [2, 48] = 0.37, *p* = 0.691, respectively). As expected, intranasal OT administration augmented plasma OT concentrations in both the TD and RAD groups (Figure 3).

### Behavioral task performance results

**Figure 4:**
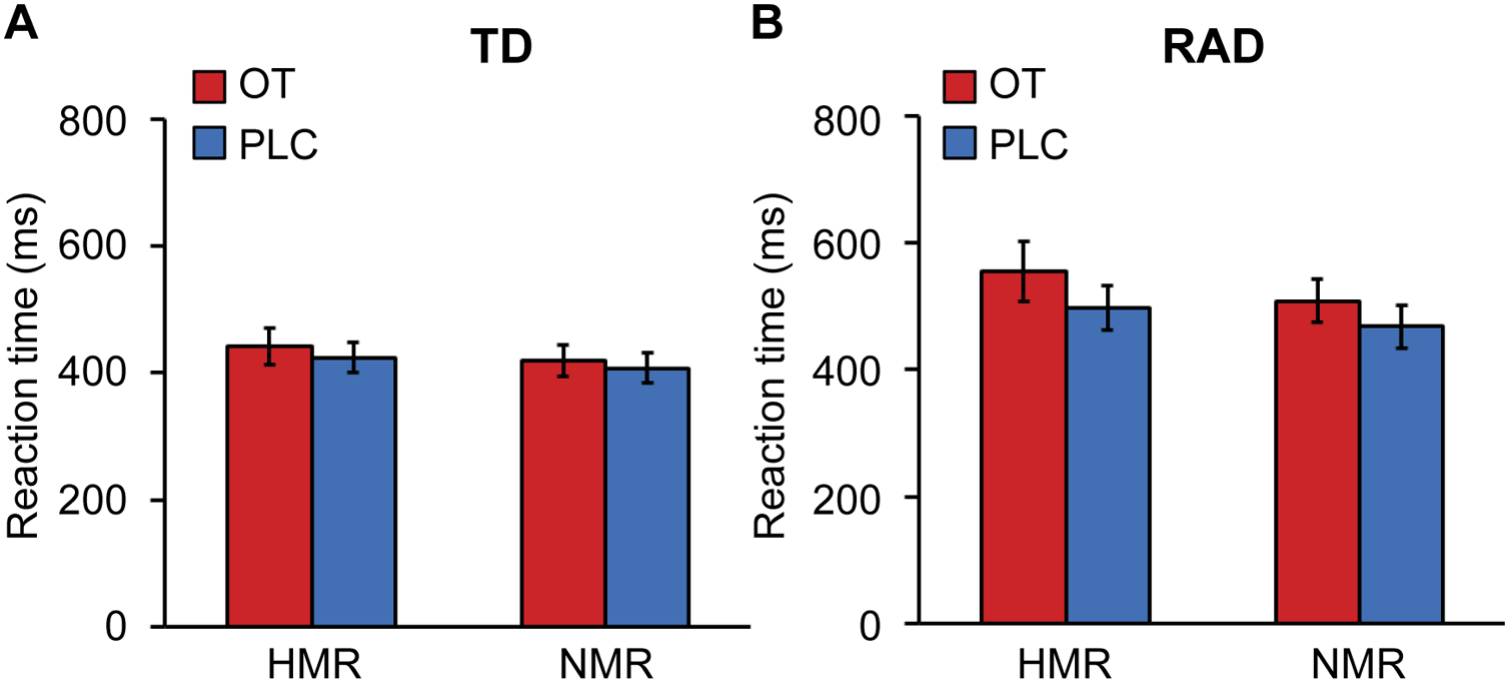
fMRI task reaction times for the reward and no-reward conditions (HMR, NMR) between (**A**) the typically developing (TD) and (**B**) reactive attachment disorder (RAD) groups under oxytocin (OT) or placebo (PLC) conditions. The plotted values represent means and standard errors of the mean. HMR, high monetary reward; NMR, no monetary reward. TD group (*n* = 27), RAD group (*n* = 24).

A three-way ANOVA was conducted to examine the effect of group (TD/RAD), treatments (OT/PLC), and reward condition (HMR/NMR) on RTs. There were significant main effects of group [*F* (1, 49) = 4.20, *p* = 0.046], treatments [*F* (1, 49) = 4.17, *p* = 0.047], and reward condition [*F* (1, 49) = 8.84, *p* = 0.0046], although they were not significant in their interactions (*p* > 0.05; see Figure 4 and Supplementary Table S1). These results indicate that the RAD group reacted significantly slower than the TD group, and the RTs during the OT condition were slower than those during the PLC condition. The RTs during the HMR condition were slower than those during the NMR condition, with no overlapping effect of the three conditions.

**Figure 5:**
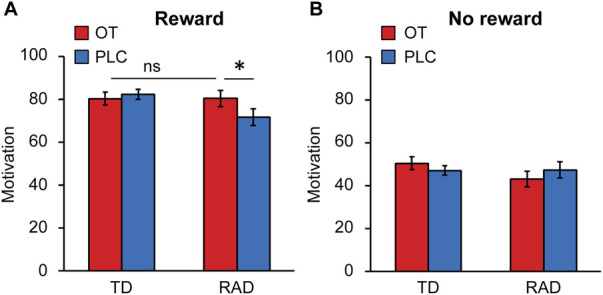
Average motivation rating on visual analog scales for (**A**) the reward or (**B**) no reward task conditions in the typically developing (TD) and reactive attachment disorder (RAD) groups after oxytocin (OT) and placebo (PLC) administration. Participants recorded their subjective motivation score from 0 (not at all) to 100 (entirely). OT significantly increased the motivation score for the reward task in the RAD group. Error bars represent standard errors of the mean (SEM). *p < 0.05. OT, oxytocin; PLC, placebo.

We investigated whether OT administration alters subjective motivation for the task. The reward conditions were significantly involved in motivation, and the VAS score was higher in the reward condition than in the no reward condition [*F* (1, 49) = 95.79, *p* < 0.001, Figures 5A,B]. Next, a two-way ANOVA was conducted to examine the effect of group (TD/RAD) and treatments (OT/PLC) on subjective motivation for the task under the high-reward condition. There were significant interactions between the effects of group and OT administration on subjective motivation for the task [*F* (1, 49) = 4.558, *p* = 0.038]. A simple main effect showed that OT administration significantly increased the VAS score in the RAD group [*F* (1, 49) = 6.082, *p* = 0.017], which was not significantly different from that in the TD group [*F* (1, 98) < 0.001, *p* = 0.987]. In contrast, OT administration did not affect the VAS score in the TD group [*F* (1, 49) = 0.306, *p* = 0.583]. Notably, this suggests that OT administration raised the motivation level for the task in the RAD group to the same level as that in the TD group. Although we investigated the relationships between OT-induced changes in RTs during the HMR condition and those in VAS scores during the reward condition, there were no significant correlations (TD group: *r* = −0.35, *p* = 0.077; RAD group: *r* = 0.17, *p* = 0.43).

### Imaging results: effect of intranasal OT/PLC administration

**Figure 6:**
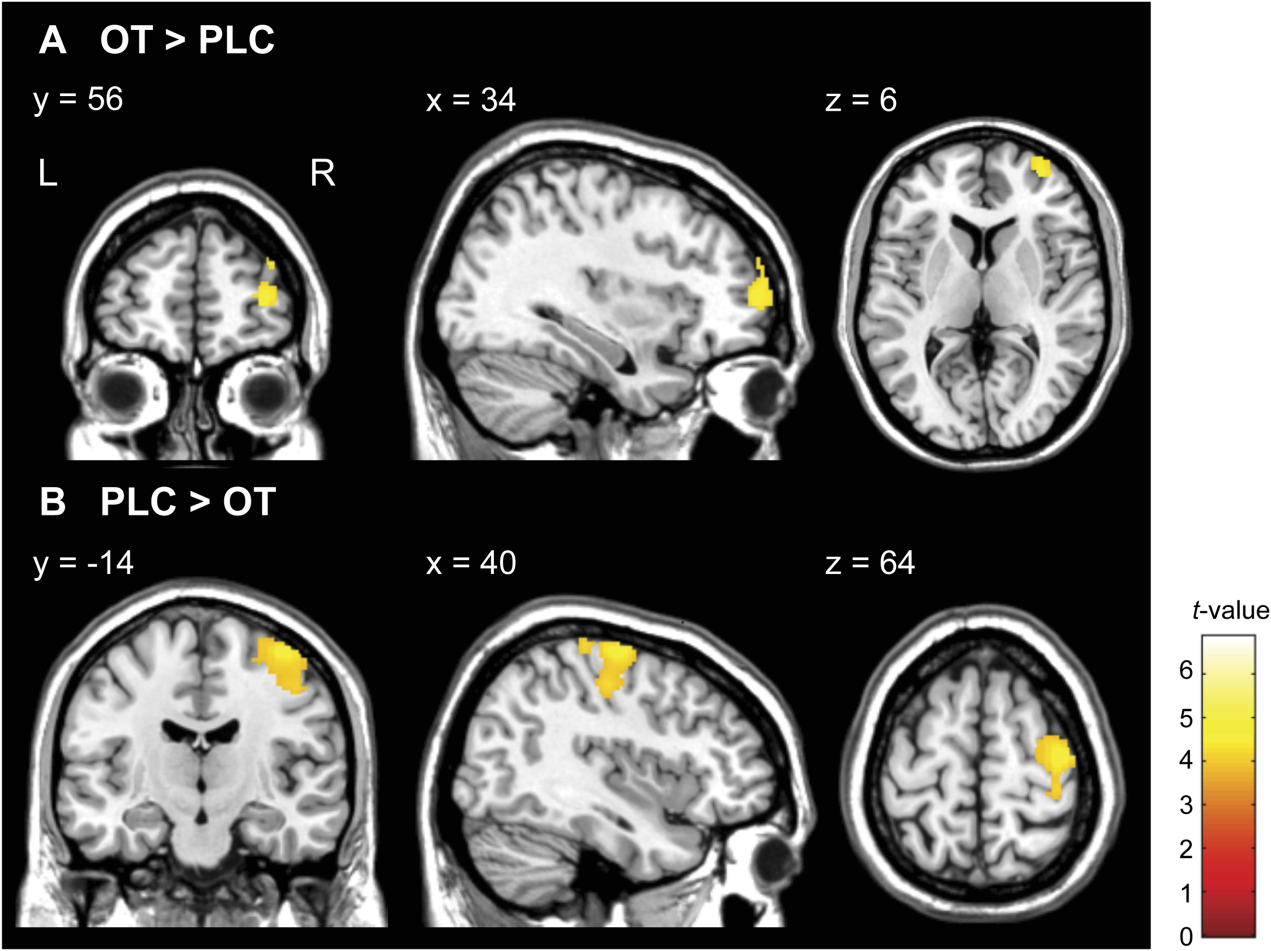
Statistical map of oxytocin (OT) effects compared to placebo (PLC) in monetary reward condition (HMR > NMR contrast) in the reactive attachment disorder (RAD) group. (**A**) OT significantly increased activity in the right middle frontal gyrus (MNI coordinates, *x* = 34, *y* = 56, *z* = 6; cluster size = 282, FWE-corrected *p* = 0.042 at the cluster level) and (**B**) reduced the activity in the right precentral gyrus (MNI coordinates, *x* = 40, *y* = −14, *z* = 64; cluster size = 934, FWE-corrected *p* < 0.001 at the cluster level) in patients with RAD. Color scales represent *t*-values. *N* = 24. HMR, high monetary reward; NMR, no monetary reward; MNI, Montreal Neurological Institute; FWE, family-wise error; OT, oxytocin; PLC, placebo; L, left; R, right.

Although there were no significant results for the interaction of group × treatments with FWE correction at the cluster level, we observed neural activation in their interactions at *p* < 0.005 uncorrected, *k* ≥ 20 (see Supplementary Table S2). We subsequently conducted two-sample *t*-tests to compare neural activations between the RAD and TD groups under PLC conditions with an FWE-corrected threshold at the cluster level. However, we did not observe the baseline differences in striatal responses between the TD and RAD groups under PLC conditions at this significance level (see Supplementary Table S3). In the one-way within-subject ANOVA on the whole-brain analysis, compared to PLC, intranasal OT significantly increased neural activation in the right middle frontal gyrus (MFG; BA 10) during the reward condition in the RAD group [Montreal Neurological Institute (MNI) coordinates, *x* = 34, *y* = 56, *z* = 6; cluster size = 282, FWE-corrected *p* = 0.042 at the cluster level, Figure 6A]. In contrast, compared to PLC, intranasal OT significantly reduced neural activation in the right precentral gyrus (BA 6) during the reward condition in the RAD group (MNI coordinates, *x* = 40, *y* = −14, *z* = 64; cluster size = 934, FWE-corrected *p* < 0.001 at the cluster level, Figure 6B). However, the RAD group showed no significant difference between PLC and OT in neural activation within any other region at this threshold (Table 2). We conducted secondary analyses to examine the possibility of moderation by medication. However, the regions of increased brain activity in the 14 medication-naïve RAD individuals were similar to those in the individuals who used medication during both the OT > PLC and PLC > OT conditions at *p* < 0.001 uncorrected, *k* ≥ 10 (see Supplementary Table S4). Therefore, medication had little to no effect on the overall results of our study. In the TD group, compared to PLC, intranasal OT neither significantly enhanced nor reduced activation in various brain regions with FWE correction at the cluster level (Table 2).

**Table 2:** Brain regions showing the effect of OT vs. PLC on functional activation during the monetary reward task (HMR > NMR contrast) in the TD and RAD groups.

Brain region	L/R	BA	MNI coordinates, mm			Cluster size	Cluster *p* (FWE-corr)	*T*-value
			x	y	z			
**TD group** (*n* = 27)								
OT > PLC			**[No significant differences]**		
PLC > OT			**[No significant differences]**		
**RAD group** (*n* = 24)								
OT > PLC								
Middle frontal gyrus	R	10	34	56	6	282	0.042	4.97
PLC > OT								
Precentral gyrus	R	6	40	−14	64	934	< 0.001	6.84

The threshold was set at *p* < 0.001 at the voxel level and FWE-corrected *p* < 0.05 at the cluster level.

OT, oxytocin; PLC, placebo; TD, typically developing; RAD, reactive attachment disorder; L/R, left/right; BA, Brodmann area; MNI, Montreal Neurological Institute.

**Figure 7:**
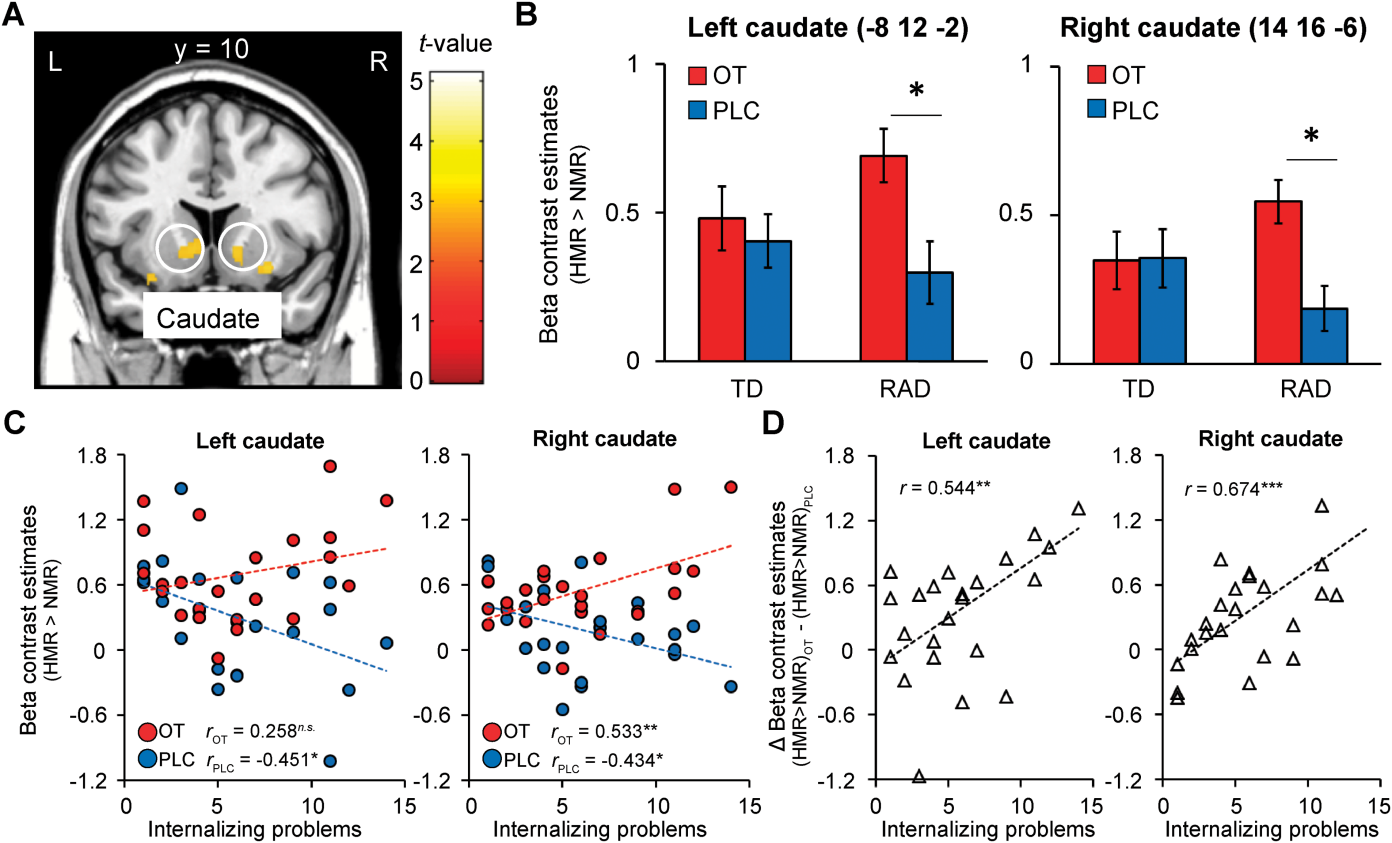
Oxytocin (OT) effects compared to placebo (PLC) on functional striatal activation during the monetary reward condition (HMR > NMR contrast) in the reactive attachment disorder (RAD) group. (**A**) OT-induced relative increases in functional activation compared to PLC in response to reward in the ventral striatum (bilateral caudate, MNI coordinates, *x* = −8, *y* = 12, *z* = −2; *x* = 14, *y* = 16, *z* = −6), and (**B**) respective beta values representing parameter estimates (±SEM); threshold of *p* < 0.005, uncorrected for multiple comparisons, *k* ≥ 20. (**C**) Internalizing problems and left and right ventral striatal responses to reward under OT and PLC in the RAD group. Scatterplot showing the correlation between symptom severity of internalizing problems (x-axis, based on SDQ internalizing problems score) and beta values of left and right ventral striatal responses during the monetary reward condition (Y-axis, contrast estimates) for the RAD group under PLC (blue dots, blue regression line: *r_PLC_* = −0.451, *p* = 0.027 and *r_PLC_* = −0.434, *p* = 0.034, respectively) and OT (red dots, red regression line: *r_OT_* = 0.258, *p* = 0.224 and *r_OT_* = 0.533, *p* = 0.007, respectively). (**D**) The effect of OT ([HMR > NMR] _OT_—[HMR > NMR] _PLC_) on striatal responses was significantly related to the severity of internalizing problems (*rΔ*_OT−PLC_ = 0.544, *p* = 0.006 and *rΔ*_OT−PLC_ = 0.674, *p* < 0.001, respectively). HMR, high monetary reward; NMR, no monetary reward; SDQ, Strengths and Difficulties Questionnaire; MNI, Montreal Neurological Institute; OT, oxytocin; PLC, placebo; L, left; R, right; SEM, standard error of the mean.

Given the limited number of studies investigating the differences in brain function between TD children and those with a history of CM and RAD, and to balance the risks of type I and II errors, we explored the potential effects of OT with a focus on the neural activity in the caudate and NAcc for reward processing during the reward condition, with a more lenient threshold of *p* < 0.005 uncorrected with a cluster size of *k* ≥ 20 voxels (64, 65). Compared to PLC, we observed small bilateral clusters of increased activation in the ventral striatum after OT administration. At this threshold, participants in the RAD group showed increased activation in the bilateral caudate nuclei (Figures 7A,B), as expected, and other regions related to the reward circuit: the superior frontal gyrus, anterior insula, orbital gyrus, and anterior cingulate cortex.

### Correlations between OT-induced neural reward responses and clinical internalizing behavior problems in the RAD group

As RAD is associated with internalizing problems, and the TD group showed minimal levels of these problems with little within-group variation (see Table 1), only the RAD group was included in these analyses. We performed a correlation analysis to investigate whether OT-induced brain activation was related to internalizing behavioral problems. In the PLC condition, the severity of internalizing problems was significantly negatively correlated with the beta values of the left and right striatal responses during the reward condition (*r_PLC_* = −0.451, *p* = 0.027 and *r_PLC_* = −0.434, *p* = 0.034, respectively; Figure 7C). After OT administration, the severity of internalizing problems was no longer significantly associated with the beta values of left striatal responses to reward (*r_OT_* = 0.258, *p* = 0.224), whereas the association between the severity of internalizing problems and the beta values of right striatal responses to reward changed significantly from negative to positive (*r_OT_* = 0.533, *p* = 0.007) (Figure 7C). Furthermore, the severity of internalizing problems was significantly positively correlated with the effect of OT ([HMR > NMR] _OT_—[HMR > NMR] _PLC_) on striatal responses (*r* Δ_OT−PLC_ = 0.544, *p* = 0.006 and *r* Δ_OT−PLC_ = 0.674, *p* < 0.001, respectively, Figure 7D).

### Correlations between OT-induced neural reward responses and behavioral task performance in the RAD group

We performed a correlation analysis to investigate whether OT-induced brain activation was related to OT-induced changes in RTs during the HMR condition and those in VAS scores during the reward condition in the RAD group. The changes in RTs and those in VAS scores were not significantly correlated with the effect of OT ([HMR > NMR] _OT_—[HMR > NMR] _PLC_) on the left and right striatal responses (*r*_ΔRT_ = −0.079, *p* = 0.71 and *r*_ΔRT_ = 0.068, *p* = 0.75, respectively; *r*_ΔVAS_ = −0.050, *p* = 0.82 and *r*_ΔVAS_ = −0.28, *p* = 0.19, respectively).

### Assessment of adverse effects of intranasal OT administration

HR, BP, and SpO_2_ showed no significant changes during the protocol. Furthermore, headache, thirst, urination, wheezing, and chest pain were neither detected nor reported by any participant.

## Discussion

To the best of our knowledge, this is the first study to use fMRI to investigate the effects of the administration of a single dose of intranasal OT on functional activation during reward processing in children and adolescents with RAD and TD individuals. We found that compared to PLC, OT administration affected motivational processing in patients with RAD at the behavioral and neurofunctional levels. Behaviorally, patients with RAD reported increased subjective motivation after OT administration and responded more slowly to reward than TD individuals. At the neurofunctional level, patients with RAD showed increased activation in the right MFG and reduced activation in the right precentral gyrus during the reward condition after OT administration. We also demonstrated, albeit at a more lenient threshold, that OT administration increased ventral striatum activation in patients with RAD. In addition, the effects of OT on the ventral striatum were significantly correlated with the severity of internalizing problems in patients with RAD.

Behaviorally, patients with RAD reported increased subjective motivation after OT administration. This is in line with findings from a previous study, in which an increase in subjective approach behavioral motivation scores was observed in patients with borderline personality disorder who had experienced severe childhood trauma after the administration of a single dose of OT (66). These findings also corroborate those of another study in which task performance increased selectively after OT administration in participants with low prosocial abilities using an incentive delay task with socially rewarding feedback (40). In this study, patients with RAD had slower RTs to the reward after OT administration. Taken together, these responses may reflect increased deliberative consideration to choose the card with higher rewards by increasing motivation. However, we observed no significant correlations between changes in VAS scores and changes in RTs, which would have been expected if these processes were causally related. This may be because the VAS score of the reward task includes subjective motivation during both HMR and LMR reward conditions.

Besides behavioral effects, our whole-brain analyses revealed a selective effect of OT administration on MFG (BA 10) activity when there was potential for obtaining a high-value monetary reward. Recent fMRI studies show that BA 10 is activated in association with monetary rewards and emotional processing, and that this lateral frontopolar area is involved in top-down cognitive control of goal-directed behaviors (67). A closely interconnected limbic and paralimbic network, including BA 10 and the ventral striatum, is considered essential in behavioral adaptation with respect to reward-induced changes in context (68). A recent fMRI study showed that intranasal OT strengthens top-down cognitive control by enhancing activity in the MFG (69), and that it activated the BA 10 region in overweight individuals during a food motivation task (70). These observations are similar to those in the present study. In our study, reduced activity in the precentral gyrus (BA 6) was detected under the reward condition after OT administration, which may also be considered expression of an adaptive process. The precentral gyrus is the primary motor area involved in finger movement (71), and considering the increased subjective motivation in patients with RAD after OT administration and their slower responses to rewards relative to that of TD individuals, patients with RAD had to inhibit their finger motor signals when carefully choosing a card owing to increased willingness to expend effort to maximize their rewards during the monetary reward tasks under OT administration.

Despite our previous findings of lower ventral striatum activation in the RAD groups compared with that in the TD groups (16, 17), herein, we did not observe the baseline differences in striatal responses between the RAD and TD groups under PLC conditions with an FWE-corrected threshold. This is consistent with previous findings of increased neural dopaminergic reward activity observed after PLC administration (72–74). Furthermore, PLC effects were significantly more favorable in children than in adults across a wide variety of psychiatric diseases (75, 76). However, our exploratory analyses showed that OT administration increased neural responses within the ventral striatum of patients with RAD. The ventral striatum is involved in approach motivation (drive toward a reward) and reward responsiveness (experience of pleasure) *via* dopaminergic and endogenous opioid activity, respectively (77). Our findings align with those of other studies showing the effects of OT in patients with PTSD, individuals exposed to trauma, patients with ASD, and healthy individuals who performed a monetary reward task (43, 45, 78, 79), suggesting that OT stimulates dopaminergic reward processing circuits. Moreover, the OT-related increase in striatal responses to non-social monetary rewards concurs with the enhancements in the mesolimbic dopaminergic reward system observed in studies investigating the OT effects on social reward (40–42). Our findings support the evidence that OT administration has broad effects on motivation and behavior, extending beyond social to non-social reward (21, 45).

Moreover, correlation analyses under placebo showed that symptom severity of internalizing problems in patients with RAD was negatively correlated with ventral striatal responses during reward condition. This is consistent with previous reports that the scores of the clinical symptoms of RAD are related to reduced striatal responses to reward (16). Interestingly, the effect of OT administration on striatal responses during the reward condition in patients with RAD was also associated with the severity of internalizing problems. This finding agrees with previous reports demonstrating that OT optimizes neural processing, especially in severely affected individuals (51); inter-individual factors modulate OT-induced reward processing (40) and selectively improve striatal responses in individuals with PTSD with high anhedonia severity (45). Children with RAD lack social and emotional reciprocity, and have difficulties regulating their emotions (10). The present findings suggest that exogenous OT promotes reward processing in RAD. It may be more beneficial for patients with RAD who report severe internalizing problems. In addition, the finding of the OT effect being stronger in the right than the left caudate may be related to the findings of our previous studies (16, 17) where the RAD group saw less activation in the right than the left caudate. However, this remains unclear and future studies are required to investigate this possibility. We observed no significant correlations between OT-induced brain activation and changes in RTs or VAS scores, which would have been expected if these processes were causally related. This may be because the VAS score of the reward task includes subjective motivation during both the HMR and LMR reward conditions. Another possibility is that the RTs of the RAD group may involve cognitive maturity given that maltreatment adversely affects cognitive function. This is in line with the findings of our previous study (16), where the RTs of the RAD group were slower than those of the TD group. An increased RT after OT administration may be a sign of increased performance monitoring, which may reflect increased deliberative consideration to choose the card with higher rewards. However, as it takes time to improve cognitive function and to evaluate the effects of intranasal OT administration on cognitive function, not only a single dose of OT but also long-term OT treatment may be necessary.

Taken together, our behavioral and neurofunctional results suggest that OT-induced increased striatal responses in patients with RAD enhance reward processing, and OT administration may increase reward expectancy and promote goal-directed behavior to obtain those rewards (45).

There has been an increase in studies examining the effects of long-term intranasal OT administration in ASD. In 11 of the 13 studies conducted over periods ranging from 4 days to 7 months, it was found that OT administration improves social abilities and skills, including social interactions and communication as well as repetitive behaviors (22). Moreover, compared to PLC, albeit based on three limited studies, long-term OT treatment appears to improve depressive and anxiety symptoms and social relationships in depressive disorders. Indeed, long-term intranasal OT-enhanced brain activity in the anterior cingulate cortex and the medial prefrontal cortex of patients with ASD has been reported (80). However, a recent placebo-controlled trial of intranasal OT therapy that included 290 children with ASD revealed no significant improvement in measures of social or cognitive functioning over 24 weeks (81). Although OT is ineffective for treating ASD symptoms, given its central role in attachment formation and prosociality, it may strengthen the neural basis of the reward system for attachment change in individuals with RAD, as in the present results. Further investigation is required to fully understand the effect of intranasal OT on symptoms other than ASD symptoms, such as attachment formation.

Recent studies indicate that intranasal OT administration induces psychosocial stress (82) and alters limbic networks, as well as limbic activation, in individuals who experienced early-life stress (83–85). These findings suggest that early social adversity can lead to decreased sensitivity to intranasal OT by changing the oxytocinergic system or its regulating genetic pathways through mechanisms such as gene methylation, thereby affecting system functioning at the OT receptor level (17, 86–90). Moreover, the opposite effects of OT administration on attachment have been found in studies on healthy adults (91) and patients with bipolar disorder (92). These studies suggest careful consideration of OT administration in individuals with a history of CM. Future studies examining the modulating effects of childhood trauma and the relationship between genetic polymorphisms, as well as the response to externally administered OT, are required to create appropriate interventions.

Plasma OT levels increased after OT but not PLC administration in the TD and RAD groups. This finding is consistent with those of previous studies and current meta-analysis-based evidence (93). Despite several studies reporting lower and higher plasma, cerebrospinal fluid, or saliva OT levels in individuals exposed to CM (30–34, 94), we did not find any significant differences in baseline plasma OT levels between the TD and RAD groups. However, some studies indicated that CM may not affect plasma OT levels (95). Future studies are needed to investigate the possible modulating factors between CM and alterations in plasma OT levels.

Our study has some limitations. First, the sample sizes were small in both groups, although they were similar to those in previous studies focusing on OT imaging (43, 45). Second, caudate activation following OT treatment increased in the RAD group when a more lenient threshold was used. Third, we did not investigate the dose-dependence of the effects of OT. Fourth, the current study was only performed in male children and adolescents for safety reasons, because there are limited studies of intranasal OT administration in female children and adolescents. Therefore, we could not account for potential sex-related differences (96). Fifth, there is a potential limitation regarding RAD diagnoses. As previously suggested (2), a child is typically given a diagnosis of “suspected” RAD, since diagnostics for it are not fully clear. Thus, detailed observations of the child's interactions are needed for a more robust diagnosis (2). Finally, as we did not control CM type, the group was relatively heterogeneous. Future studies are needed to address these limitations.

## Conclusion

Our study provides the first evidence that a single dose of OT administered intranasally increased subjective motivation and enhanced neural reward activity in male children and adolescents with RAD. Furthermore, our findings suggest that OT administration increases reward processing and has the potential to promote motivational behaviors; therefore, it may be beneficial for patients with RAD who report severe internalizing problems. These results provide important insights toward investigating the therapeutic potential and effectiveness of combining OT administration with child-caregiver psychotherapy sessions for RAD (97–100). Future clinical trials should consider whether long-term OT administration is beneficial for patients with RAD.

## Data Availability

The raw data supporting the conclusions of this article will be made available by the authors, without undue reservation.
