# Supplementary material for: Effects of intranasal oxytocin on neural reward processing in children and adolescents with reactive attachment disorder: A randomized controlled trial
Source: Front Child Adolesc Psychiatry. 2023 Jan 10;1:1056115. doi: 10.3389/frcha.2022.1056115 (PMC11748893; doi:10.3389/frcha.2022.1056115)
Supplement: Supplementary file 1 [file Table1.docx]

***Supplementary Material***

**Effects of intranasal oxytocin on neural reward processing in children and adolescents with reactive attachment disorder: 　　　　　　　　　a randomized controlled trial**

Shinichiro Takiguchi^1, 2^, Kai Makita^3^, Takashi X. Fujisawa^2, 3^, Shota Nishitani^2, 3^, Akemi Tomoda^1, 2, 3^*

*** Correspondence:** Akemi Tomoda: atomoda@u-fukui.ac.jp

# Supplementary Materials and Methods

## Participants

Some of the participants with RAD were living in a stable placement (i.e., child welfare facility), but not with their biological parents—for detailed information about child welfare services in Japan, see Suzuki and Tomoda, 2015 (1). The Edinburgh Handedness Inventory (2) was administered to both the TD and RAD groups.

## Study design and procedure: sample size and intranasal oxytocin administration

The required sample size of more than 20 participants per group (i.e., male patients with RAD and TD controls) was calculated using a statistical power analysis (3). A minimum of 12 subjects were needed to insure 80% power at α =0.05 at the single-voxel level. In fact, 14–23 participants with ASD or other psychiatric disorders were included in previous task-related fMRI studies that reported significant neural activations in fMRI tasks after single-dose oxytocin (OT) administration (4-7). Considering these studies, we set the current sample size at more than 20 (within-subject design).

A 24 IU dose has been the most frequently used dose in previous studies, and intranasal OT has shown no severe side effects when delivered in doses of 18–40 IU (8,9), although mild side effects have been reported in children (10).

## Measurement of plasma OT concentration

Before blood collection, participants were resting (no eating or drinking) for approximately 30 min. Plasma OT concentrations were determined using an enzyme immunoassay Oxytocin ELISA kit (Enzo Life Sciences, Inc., Farmingdale, NY, USA). This practice ensured that plated samples contained twofold concentrated OT, a sufficiently high quantity to be read above the detection limit (15.0 pg/mL).

## Monetary reward tasks

The task and stimuli in this study were the same as those used in our previous study (11) and have been described as follows:

All participants were encouraged to try to earn as much money as possible and were told that one session would be randomly chosen at the end of the experiment and that their earnings in that session would be given to them. During each trial (3 s), participants were presented with three cards labeled “A,” “B,” and “C” within 2 s; they were asked to choose one of the three cards by pressing a button. Immediately after pressing the button, the chosen card was highlighted and the outcome was displayed for 1 s. Each card was randomly associated with 0, 30, or 60 yen. Three conditions of eight trials (24 s) were performed. Unknown to participants, the total reward was predetermined. In the high monetary reward (HMR) condition, participants earned an average of 330 yen (range, 270–390 yen), which was consistently higher than the expected value of the eight reward trials (240 yen). In the low monetary reward (LMR) condition, participants earned an average of 150 yen (range, 90–210 yen), which was consistently lower than the expected value. In the no monetary reward (NMR) condition, the outcome presented was always ‘‘XXX’’ to control for effects other than reward level. The NMR condition or a fixation rest condition (24 s) was always inserted between the two reward conditions. All participants completed a practice task for 2 min before undergoing MRI. During MRI, participants performed four sessions. Each session consisted of four blocks of each of the four conditions (HMR, LMR, NMR, and fixation rest) within which the HMR and LMR conditions were ordered differently and therefore lasted 6 min 24 s (4 blocks × 4 conditions × 24 s per block). The order of the four sessions was counterbalanced across participants.

## fMRI acquisition and analysis

Data acquisition, pre-processing, and first-level analysis have been described in our previous study (11) as follows:

All scans were acquired using a 3-T MR scanner (Discovery MR 750; General Electric Medical Systems, Milwaukee, WI, USA) with a 32-channel head coil. For functional imaging, a series of 528 volumes (132 volumes per session) was acquired using an interleaved T2-weighted, gradient echo, echo planar imaging (EPI) sequence. Each volume consisted of 44 transaxial slices (thickness, 3.0 mm between slices) that included the entire cerebrum and cerebellum (repetition time [TR], 3000 ms; echo time [TE], 25 ms; flip angle [FA], 90°; field of view [FOV], 192 mm; in-plane matrix size, 64 × 64 pixels, voxel dimensions, 3.0 × 3.0 × 3.0 mm; slice gap, 0 mm).

Data were analyzed using SPM12 (The Wellcome Trust Centre for Neuroimaging, London, UK) implemented in MATLAB 2020a (Mathworks, Natick, MA, USA). Following realignment, all images were normalized to the SPM12 (EPI) image template. The anatomically normalized EPI data were spatially smoothed in three dimensions using an 8 mm full-width half-maximum Gaussian kernel.

At the first level, individual task-related activation was evaluated. Three regressors for each condition, HMR, LMR, and NMR, were modelled at the onset of each block (duration, 24 s), which were convolved with a canonical hemodynamic response function to obtain the expected task-related signal change. The weighted sum of the parameters estimated during individual analyses consisted of “contrast” images. Global signal changes were utilized to remove confounding factors, such as scanner gain. For the statistical analyses, motion parameters were modeled as regressors of no interest using the six parameters (three displacements and three rotations) obtained by the rigid-body realignment procedure. The data were high-pass filtered with a cut-off period of 128 s to remove low-frequency signal drifts. An autoregressive model was used for whitening the residuals to meet general linear model assumptions.

# Supplementary Tables

***Behavioral task performance results***

**Table S1: Results of three-way ANOVA** **of the RT.**

| Source | Sum of Squares | df | Mean Square | *F* | *p* |  |
| --- | --- | --- | --- | --- | --- | --- |
| A (RAD/TD) | 353956.26 | 1 | 353956.26 | 4.20 | 0.046 | * |
| B (OT/PLC) | 50996.63 | 1 | 50996.63 | 4.17 | 0.047 | * |
| C (HMR/NMR) | 41325.83 | 1 | 41325.83 | 8.84 | 0.0046 | *** |
| A**×**B | 14806.99 | 1 | 14806.99 | 1.20 | 0.28 |  |
| A**×**C | 4109.23 | 1 | 4109.23 | 0.88 | 0.35 |  |
| B**×**C | 1661.73 | 1 | 1661.73 | 0.51 | 0.48 |  |
| A**×**B**×**C | 382.34 | 1 | 382.34 | 0.12 | 0.73 |  |
| Error | 158428.26 | 49 | 3233.23 |  |  |  |
| A: group, B: treatments, C: reward condition | |  |  | **p*<0.05, ****p*<0.005 | | |

RT, reaction time; TD, typically developing; RAD, reactive attachment disorder; OT, oxytocin; PLC, placebo; HMR, high monetary reward; NMR, no monetary reward.

***Imaging results: effect of intranasal OT/PLC administration***

**Table S2: Peak brain activation for the flexible factorial design in the whole brain general linear model analysis: Interaction of group (TD/RAD) × treatment (OT/PLC).**

| Brain region | L/R | MNI coordinates, mm | | | *F* | *p* (unc) |
| --- | --- | --- | --- | --- | --- | --- |
|  |  | x | y | z |  |  |
| **Interaction group (TD/RAD) ×** **treatment (OT/PLC)** | | | | | | |
| Middle frontal gyrus | R | 44 | 46 | 22 | 17.79 | 0.000 |
| Middle frontal gyrus | L | -38 | 56 | 16 | 13.71 | 0.001 |
| Precentral gyrus | R | 38 | -8 | 64 | 14.58 | 0.000 |
| Precentral gyrus | L | -42 | -8 | 60 | 14.74 | 0.000 |
| Angular gyrus | L | -40 | -68 | 46 | 13.59 | 0.001 |
| Superior frontal gyrus medial segment | L | -2 | 36 | 54 | 13.18 | 0.001 |
| Postcentral gyrus | L | -40 | -36 | 58 | 12.35 | 0.001 |
| Superior frontal gyrus | L | -18 | 50 | -10 | 12.22 | 0.001 |
| Superior parietal lobule | R | 14 | -50 | 72 | 11.24 | 0.002 |
| Superior temporal gyrus | L | -64 | -22 | 6 | 11.10 | 0.002 |
| Supplementary motor cortex | R | 4 | -10 | 54 | 10.92 | 0.002 |
| Precuneus | L | -12 | -46 | 56 | 10.77 | 0.002 |

All results reported at *p*<0.005 uncorrected, *k*≥20.

TD, typically developing; RAD, reactive attachment disorder; OT, oxytocin; PLC, placebo; L, left; R, right; MNI, Montreal Neurological Institute

**Table S3. Comparison of the activity in brain regions showing the effect of PLC during the monetary reward task (HMR > NMR contrast) between the TD and RAD groups.**

| Brain region | L/R | MNI coordinates, mm | | | Cluster size | Cluster *p* (FWE-corr) | *T*-value |
| --- | --- | --- | --- | --- | --- | --- | --- |
|  |  | x | y | z |  |  |  |
| **TD group > RAD group** |  |  | | | |  |  |
| [No significant differences] | | | | | | | |
| **RAD group < TD group** |  |  | | | |  |  |
| [No significant differences] | | | | | | | |

The threshold was set at *p* < 0.001 at the voxel level and FWE-corrected *p* < 0.05 at the cluster level.

TD, typically developing; RAD, reactive attachment disorder; PLC, placebo; HMR, high monetary reward; NMR, no monetary reward; L, left; R, right; MNI, Montreal Neurological Institute; FWE, family-wise error

**Table S4: Brain regions showing the effect of OT vs. PLC on functional activation during the monetary reward task (HMR > NMR contrast) in the medication-naïve RAD groups (n = 14).**

| Brain region | L/R | MNI coordinates, mm | | | Cluster size | Cluster *p* (FWE-corr) | *T*-value |
| --- | --- | --- | --- | --- | --- | --- | --- |
|  |  | x | y | z |  |  |  |
| OT > PLC |  |  |  |  |  |  |  |
| Middle frontal gyrus | R | 36 | 60 | 14 | 36 | 0.873 | 5.51 |
| Middle frontal gyrus | L | -28 | 54 | 14 | 19 | 0.972 | 4.27 |
| PLC > OT |  |  |  |  |  |  |  |
| Precentral gyrus | R | 42 | -26 | 68 | 252 | 0.030 | 5.73 |

All results reported at *p*<0.001 uncorrected, *k*≥10.

RAD, reactive attachment disorder; OT, oxytocin; PLC, placebo; HMR, high monetary reward; NMR, no monetary reward; L, left; R, right; MNI, Montreal Neurological Institute; FWE, family-wise error

# Supplementary references

1. Suzuki H, Tomoda A. Roles of attachment and self-esteem: impact of early life stress on depressive symptoms among Japanese institutionalized children. BMC Psychiatry (2015) 15:8. doi: 10.1186/s12888-015-0385-1

2. Oldfield RC. The assessment and analysis of handedness: the Edinburgh inventory. Neuropsychologia (1971) 9:97-113. doi: 10.1016/0028-3932(71)90067-4

3. Desmond JE, Glover GH. Estimating sample size in functional MRI (fMRI) neuroimaging studies: statistical power analyses. J Neurosci Methods (2002) 118:115-28. doi: 10.1016/S0165-0270(02)00121-8

4. Domes G, Heinrichs M, Kumbier E, Grossmann A, Hauenstein K, Herpertz SC. Effects of intranasal oxytocin on the neural basis of face processing in autism spectrum disorder. Biol Psychiatry (2013) 74:164-71. doi: 10.1016/j.biopsych.2013.02.007

5. Aoki Y, Yahata N, Watanabe T, Takano Y, Kawakubo Y, Kuwabara H, et al. Oxytocin improves behavioural and neural deficits in inferring others' social emotions in autism. Brain (2014) 137:3073-86. doi: 10.1093/brain/awu231

6. Kruppa JA, Gossen A, Oberwelland Weiß E, Kohls G, Großheinrich N, Cholemkery H, et al. Neural modulation of social reinforcement learning by intranasal oxytocin in male adults with high-functioning autism spectrum disorder: a randomized trial. Neuropsychopharmacology (2019) 44:749-56. doi: 10.1038/s41386-018-0258-7

7. Frijling JL, van Zuiden M, Koch SB, Nawijn L, Veltman DJ, Olff M. Intranasal oxytocin affects amygdala functional connectivity after trauma script-driven imagery in distressed recently trauma-exposed individuals. Neuropsychopharmacology (2016) 41:1286-96. doi: 10.1038/npp.2015.278

8. Fragkaki I, Glennon JC, Cima M. Salivary oxytocin after oxytocin administration: examining the moderating role of childhood trauma. Biol Psychol (2020) 154:107903. doi: 10.1016/j.biopsycho.2020.107903

9. MacDonald E, Dadds MR, Brennan JL, Williams K, Levy F, Cauchi AJ. A review of safety, side-effects and subjective reactions to intranasal oxytocin in human research. Psychoneuroendocrinology (2011) 36:1114-26. doi: 10.1016/j.psyneuen.2011.02.015

10. DeMayo MM, Song YJ, Hickie IB, Guastella AJ. A review of the safety, efficacy and mechanisms of delivery of nasal oxytocin in children: therapeutic potential for autism and Prader-Willi syndrome, and recommendations for future research. Pediatr Drugs (2017) 19:391-410. doi: 10.1007/s40272-017-0248-y

11. Nishitani S, Fujisawa TX, Hiraoka D, Makita K, Takiguchi S, Hamamura S, et al. A multi-modal MRI analysis of brain structure and function in relation to OXT methylation in maltreated children and adolescents. Transl Psychiatry (2021) 11:589. doi: 10.1038/s41398-021-01714-y
